# Preclinical and clinical developments for combination treatment of influenza

**DOI:** 10.1371/journal.ppat.1010481

**Published:** 2022-05-12

**Authors:** Paulina Koszalka, Kanta Subbarao, Mariana Baz

**Affiliations:** 1 WHO Collaborating Centre for Reference and Research on Influenza at The Peter Doherty Institute for Infection and Immunity, Melbourne, Australia; 2 Biomedicine Discovery Institute & Department of Microbiology, Monash University, Clayton, Australia; 3 Department of Microbiology and Immunology, University of Melbourne at The Peter Doherty Institute for Infection and Immunity, Melbourne, Australia; University of Pittsburgh, UNITED STATES

## Abstract

Antiviral drugs are an important measure of control for influenza in the population, particularly for those that are severely ill or hospitalised. The neuraminidase inhibitor (NAI) class of drugs, including oseltamivir, have been the standard of care (SOC) for severe influenza illness for many years. The approval of drugs with novel mechanisms of action, such as baloxavir marboxil, is important and broadens potential treatment options for combination therapy. The use of antiviral treatments in combination for influenza is of interest; one potential benefit of this treatment strategy is that the combination of drugs with different mechanisms of action may lower the selection of resistance due to treatment. In addition, combination therapy may become an important treatment option to improve patient outcomes in those with severe illness due to influenza or those that are immunocompromised. Clinical trials increasingly evaluate drug combinations in a range of patient cohorts. Here, we summarise preclinical and clinical advances in combination therapy for the treatment of influenza with reference to immunocompromised animal models and clinical data in hospitalised patient cohorts where available. There is a wide array of drug categories in development that have also been tested in combination. Therefore, in this review, we have included polymerase inhibitors, monoclonal antibodies (mAbs), host-targeted therapies, and adjunctive therapies. Combination treatment regimens should be carefully evaluated to determine whether they provide an added benefit relative to effectiveness of monotherapy and in a variety of patient cohorts, particularly, if there is a greater chance of an adverse outcome. Safe and effective treatment of influenza is important not only for seasonal influenza infection, but also if a pandemic strain was to emerge.

## Introduction

### What is the role of antiviral drugs for influenza?

Influenza viruses belong to the Orthomyxoviridae family, are divided into 4 types (A, B, C, and D), and cause a respiratory illness in humans [1]. Seasonal epidemics cause significant levels of morbidity and mortality worldwide, and pandemics can occur at irregular intervals and further exacerbate the burden on human health (1918, 1957, 1968, and 2009). Spillover of zoonotic infections in humans, including H5N1 and H7N9, are also a major concern [2]. Vaccination and antiviral drugs are 2 control measures for influenza; annual vaccination programs for influenza are well established in many countries; however, vaccine formulation must anticipate the virus that will circulate in an upcoming influenza season based on influenza activity in the reciprocal hemisphere of the world. Antigenic mismatch between circulating viruses and vaccine strains can occur [3] and vaccine responses elicited in high-risk groups, including the elderly and immunocompromised persons, may be inadequate [4,5]. Vaccine production to novel influenza strains is estimated to require 6 months; during this time, antiviral drugs may be of significant public health benefit. Antiviral drugs are important for the treatment of patients that are critically ill or hospitalised or can be utilised to treat spillover zoonotic infections [6].

### Currently approved antiviral drugs for influenza

Three classes of antiviral drugs are currently approved for the treatment of influenza. The adamantanes target the M2 proton channel of influenza A viruses and were first identified in 1964 but have limited clinical use as nearly 100% of viruses have an M2 substitution (S31N) that confers resistance [7].

The neuraminidase inhibitors (NAIs) inhibit the enzymatic function of the neuraminidase (NA) protein and were formulated with rational drug design. The NAIs include oseltamivir, zanamivir, peramivir and laninamivir; these have been licensed since the early 2000s and are widely used to treat influenza [8,9]. The NA/H275Y amino acid substitution is a major path for viral resistance to oseltamivir.

Baloxavir marboxil is a polymerase inhibitor and targets the endonuclease function of the PA protein [10]. The drug was first licensed in 2018 in Japan and the United States and continues to be licensed in many countries [11,12]. In clinical trials, baloxavir has been shown to be more effective than oseltamivir at reducing viral shedding in patients [13].

### Why are we interested in antiviral combinations?

#### Reduced selection of antiviral resistance mutations

Resistant viruses have been detected for all licensed influenza antivirals and can lead to poor clinical outcomes in patients [14,15], viral spread in geographic clusters [16,17], or if sufficiently fit can circulate globally and limit the usefulness of antiviral treatment [18]. Combination antiviral therapies for human immunodeficiency virus (HIV) and hepatitis C virus (HCV) have been shown to decrease the selection of resistant viruses, as drug combinations with different mechanisms of action should remain effective if viruses with drug resistance emerge [19–22]. Combination therapy may be a useful option for immunocompromised or severely ill patients [23–25], as antiviral therapy in these patients over extended periods (several weeks or months) can increase the probability of selecting for resistant viruses [26–30].

#### Correlation of in vitro synergy with clinical outcomes

In preclinical studies, the effect of drug interactions can be defined as antagonistic, additive, or synergistic. Antagonistic or synergistic effects are lower or greater, respectively, than the sum of the drug effects alone—which would be additive [31]. Combinations of antiviral drugs may show synergy on the reduction of viral replication in cell-based or preclinical studies; however, in clinical trials, the primary endpoint of antiviral drug treatment is often based on time to the improvement of clinical symptoms while reduction in viral titre is a secondary endpoint. The correlation of symptoms and viral load in patients relative to monotherapy is an important consideration as higher viral loads can be associated with increased severity of influenza symptoms and the converse with lower viral loads [32–35].

#### Reduction in severe outcomes or complications of infection

In patients at risk of complications due to influenza, severe outcomes or death may be averted with antiviral treatment. For example, studies in pregnant women and hospitalised children have shown that timely antiviral treatment could greatly reduce the length of hospital stay or progression to severe disease [36,37]. Drug combinations are increasingly evaluated with standard of care (SOC), which is important to understand how severe outcomes with combination therapy compare to monotherapy [38–40].

#### Reduced drug dose that results in decreased drug toxicity or adverse effects

Drug interactions can occur at several stages including drug absorption, elimination, or metabolism. Adverse interactions between drugs can lead to undesirable effects, and therefore, drug–drug interactions must be carefully assessed [41]. Dose-related drug effects may be reduced by combining antiviral drugs if a lower dose of either drug can be administered [42].

### Why is it important to consider different population groups for clinical trials of antiviral treatments?

High-risk groups include the elderly, pregnant women, patients on immunosuppressive treatments, and individuals with chronic conditions such as obesity, asthma, and diabetes. Prolonged viral replication and severe disease may increase the risk of complications and death in these populations. Diverse medical histories pose a challenge for the design of randomised controlled trials in hospitalised patients, and it may be considered unethical to withhold the SOC from a severely ill patient; investigational drugs may therefore be added to SOC and compared to SOC alone [43].

### Purpose of this review

In this review, we summarise the available data on drugs used in combination to treat influenza. Due to limited clinical use, the adamantanes are excluded and NAI combinations with other NAI drugs are not discussed. However, NAIs are often SOC and combined with investigational antivirals and discussed in this context. Some excellent reviews have been published on adamantanes and NAIs in combination therapies [44–47]. This review will summarise preclinical data, including immunosuppressed animal models, and clinical trials including in high-risk patient cohorts for direct-acting influenza antivirals, monoclonal antibodies (mAbs), and host-targeted therapies.

### Viral polymerase inhibitors

There are 3 major polymerase inhibitors in clinical development, baloxavir (formerly S-033188), which inhibits the polymerase acidic (PA) protein [48]; favipiravir (formerly T-705), which is a purine nucleoside analogue that blocks the function of polymerase basic protein 1 (PB1) [49]; and pimodivir, which inhibits the polymerase basic protein 2 (PB2) (Table 1) [50]. Baloxavir is active against influenza A and B viruses, and pimodivir is only active against influenza A due to structural differences, whereas favipiravir has broad-spectrum antiviral activity against RNA viruses including filoviruses, arenaviruses, coronaviruses, and bunyaviruses [51–53].

**Table 1:** Summary of antiviral drugs and adjunctive therapies used in combination treatment in clinical trials.

Drug name	Mechanism of action	Route of administration	Typical monotherapy dose	Clinical trial/licensed	Drug combinations tested in clinical trials
***Polymerase inhibitors***					
Baloxavir marboxil	PA endonuclease inhibitor	Oral	40-mg single dose (weight < 80 kg) or 80 mg single dose (weight > 80 kg)	Licensed	SOC NAIs: hospitalised participants with severe influenza (NCT03684044)
Favipiravir	Purine nucleoside analogue	Oral (intravenous under development)	Day 1: 1,800 mg BID Days 2 to 5: 800 mg BID	Restricted licensure	Oseltamivir: pharmacokinetics for severe influenza (NCT03394209) Oseltamivir: prospective study in patients critically ill with influenza
Pimodivir	PB2 inhibitor	Oral (intravenous under development)	600 mg; BID for 5 days	III	Oseltamivir: pharmacokinetic study in healthy volunteers (NCT02262715) Oseltamivir: patients with uncomplicated influenza (NCT02342249) Oseltamivir: hospitalised adolescents (NCT03376321) Oseltamivir: high-risk outpatients (NCT03381196)
***mAbs***					
VIS410	Influenza A HA stem	Intravenous	Single fixed dose	II	Oseltamivir: hospitalised adults with influenza A infection requiring oxygen support (NCT03040141)
MHAA4549A	Influenza A HA stalk	Intravenous	Single fixed dose	II	Oseltamivir: patients with severe influenza A infection (NCT02293863)
MEDI8852	Influenza A HA stem	Intravenous	Single fixed dose	IIa	Oseltamivir: patients with uncomplicated influenza (NCT02603952)
***Anti-inflammatory***					
Celecoxib	Nonsteroidal anti-inflammatory drug	Oral	200 mg; once a day	III (for influenza treatment)	Oseltamivir: severe influenza A infection (NCT02108366)
Diltiazem	Calcium channel blocker	Oral	60 mg; TID	II (for influenza treatment)	Oseltamivir: severe influenza A infection (NCT03212716)
***Host targeted***					
Nitazoxanide	HA glycosylation	Oral	300 mg; BID	III	Oseltamivir: acute uncomplicated influenza (NCT01610245)
***Antivirals + antibiotics***					
Azithromycin	Antibiotic	Oral	500 mg; TID	Not listed	Oseltamivir: high-risk seasonal influenza (UMIN000005371)
Clarithromycin	Antibiotic	Oral	500 mg; once a day	II (for influenza treatment)	Naproxen and Oseltamivir: hospitalised pediatric patients (NCT04315194)

BID, bis in die (twice a day); mAb, monoclonal antibody; NAI, neuraminidase inhibitor; SOC, standard of care; TID, ter in die (3 times a day).

For baloxavir, resistant viruses have been detected in adult patients (>12 years: 1.1% to 14.6%) and children (<12 years: 18.9% to 52.2%) and is most commonly associated with PA/I38 amino acid substitutions, the most frequent variant is PA/I38T [54]. Several resistant viruses to pimodivir were identified in clinical trials including S324C, K376R, F325L, and M431L/R/V in the PB2 protein [55,56].

### Baloxavir in vitro and preclinical studies

In vitro and in vivo synergy of baloxavir and the NAIs has been shown for A(H1N1), A(H1N1pdm09), A(H3N2), and influenza B viruses [57,58]. Fukao and colleagues studied delayed treatment (96 hours postinfection) in a mouse model with baloxavir, oseltamivir, or the combination of both drugs using a lethal dose of influenza A/PR/8/34 [58]. Combination therapy was just as effective as monotherapy with 15 or 50 mg/kg BID (twice a day) of baloxavir for reduction of viral lung titres and mouse lung pathology [58]. However, for reduction in mortality baloxavir (0.5 mg/kg) + oseltamivir (either 10 or 50 mg/kg) was more effective than monotherapy.

In an immunodeficient mouse model, antiviral treatment with favipiravir, baloxavir, or oseltamivir was commenced 48 hours postinfection with a mouse-adapted A/Switzerland/9715293/2013 (H3N2) [59]. Significant weight loss was observed with oseltamivir (20 mg/kg BID) and favipiravir (100 mg/kg BID) monotherapy, combination treatment prevented weight loss similarly to baloxavir (20 mg/kg BID) monotherapy. Baloxavir monotherapy, oseltamivir + baloxavir and oseltamivir + favipiravir + baloxavir reduced mortality equally, but oseltamivir and favipiravir monotherapy offered no protection [59]. Mouse viral lung titres reduced the most with treatments that contained baloxavir (including monotherapy); however, favipiravir or oseltamivir were not synergistic in reducing lung titres when combined with baloxavir [59]. Viruses recovered from 50% of the mice (2 of 4) treated with oseltamivir had an NA/E119V substitution, but this was not identified with combination treated mice [59].

A serial passage experiment in mice compared the effect of treatment with baloxavir monotherapy, oseltamivir monotherapy or baloxavir + oseltamivir in combination on selection of resistant viruses [60]. With oseltamivir monotherapy, no drug-resistant viruses were identified and for baloxavir monotherapy 2 of 3 mice had viruses with PA/I38X substitutions. For baloxavir + oseltamivir combinations, an NA/N274Y substitution was identified, but no amino acid substitutions were noted in the PA protein [60].

### Baloxavir clinical studies

The combination of baloxavir with SOC NAIs is of particular interest as both drugs are licensed and available in many countries (Table 1). No significant adverse effects or meaningful drug–drug interactions were observed in 18 healthy patients when baloxavir or oseltamivir monotherapy were compared to baloxavir + oseltamivir combination therapy. [61]. A retrospective study in Japan measured reduction of mortality in patients hospitalised with influenza and treated with peramivir + baloxavir [62]. The mortality rate was 4.5% in the peramivir only treatment group (132 patients) and none in the peramivir + baloxavir group (10 patients). A larger multicentre study would be required to confirm this; however, this study suggests that this combination may reduce patient mortality.

A Phase III clinical trial (Flagstone; NCT03684044) conducted in hospitalised patients with severe influenza compared the combination of SOC NAIs with baloxavir or placebo (Table 2) [40]. Combination treatment did not add clinical benefit compared to NAI alone; the median time to clinical improvement was 97.5 hours for baloxavir + NAI and 100.2 hours for SOC [40]. A secondary endpoint of the study was time to cessation of viral shedding; the median time was 23.9 hours for baloxavir + NAI and 63.7 hours for SOC, respectively [40]. Dual drug resistance (NA/H275Y + PA/I38T) emerged in viruses isolated from 2 immunocompromised patients treated with an NAI (oseltamivir) in combination with baloxavir [40].

**Table 2:** Clinical trials with antiviral or other drugs used in combination for the treatment of influenza.

Drug combination	Clinical trial phase	Target population/eligibility criteria (actual enrollment)	Treatment intervention	Primary outcome measures	ClinicalTrials.gov NTC identifier
Baloxavir + SOC NA (oseltamivir, zanamivir, or peramivir)	III	Hospitalised patients with severe influenza, ages 12 years or older (*n* = 373)	At least 2 doses of baloxavir marboxil (day 1 and 4), third dose on day 7 for participants that had not improved based on predefined criteria. Alternatively, a placebo comparator is administered on the same schedule as baloxavir marboxil. Baloxavir marboxil or placebo are given in combination with local SOC NAI (oseltamivir, zanamivir, or peramivir)	Time to clinical improvement based on hospital discharge or NEWS2	NCT03684044
Favipiravir + oseltamivir	II	Patients with severe influenza, aged 18 years and older (*n* = 34)	For the low-dose group, favipiravir 1,600-mg BID for 1 day and then on days 2 to 9 600-mg BID in combination with oseltamivir 75-mg BID for 10 days. For the high-dose group, favipiravir 1,800-mg BID for 1 day and then on days 2 to 9 800-mg BID in combination with oseltamivir 75-mg BID for 10 days	The number of patients that reach the minimum plasma concentration of favipiravir	NCT03394209
VX-787 (pimodivir) + oseltamivir	I	Healthy participants, aged 18 to 55 years (*n* = 38)	VX-787 (pimodivir), or a matching placebo, 600-mg BID on day 1 to 4, and 1 dose 600 mg on day 5. Compared to oseltamivir, 75-mg BID on day 1 to 4, 1 dose 75 mg on day 5. VX-787 and oseltamivir given in combination at doses and times described for monotherapy	To assess potential drug–drug interaction between VX-787 administered with oseltamivir in healthy participants	NCT02262715
VX-787 (pimodivir) + oseltamivir	IIb	Adults with uncomplicated seasonal influenza, aged 16 to 84 years (*n* = 292)	VX-787 (pimodivir) 300-mg BID with placebo BID for 5 to 6 days. VX-787 600-mg BID with placebo BID for 5 to 6 days. VX-787 600-mg BID in combination with oseltamivir 75-mg BID for 5 to 6 days	AUC of nasal viral load on day 8, measured by qRT-PCR	NCT02342249
Pimodivir + oseltamivir	II	Adult and elderly patients hospitalised with influenza, aged 18 to 85 (*n* = 102)	JNJ-63623872 (pimodivir) 600-mg BID, or a matching placebo, in combination with oseltamivir 75-mg BID for 7 days	Maximum and minimum observed plasma concentration of pimodivir and area under the plasma concentration–time curve	NCT02532283
Pimodivir + SOC	III	Adolescent, adult, and elderly patients hospitalised with influenza A, ages 13 to 85 (*n* = 334)	Pimodivir 600-mg BID for 5 days, or a matching placebo, on days 1 through 5 and 1 dose day 6 administered with local SOC	Clinical status based on number of participants with hospital recovery scale on day 6	NCT03376321
Pimodivir + SOC	III	Adolescent, adult, and elderly participants that are not hospitalised with influenza A, but at risk of complications, age 13 to 85 years (*n* = 553)	Pimodivir 600-mg BID for 5 days, or a matching placebo, on days 1 through 5 and 1 dose day 6 administered with local SOC	Time to resolution of 7 primary influenza-related symptoms as assessed by the PRO and measure flu-intensity and impact questionnaire (Flu-iiQ)	NCT03381196
VIS410 + oseltamivir	II	Hospitalised patients infected with Influenza A that require oxygen, age 18 years or older (*n* = 89)	Either “low dose” or “high dose” or matching placebo of VIS410 intravenously in combination with oseltamivir	(1) Clinical outcome assessed by ordinal day 7 scores between treatment groups. (2) Safety and tolerability of intravenous doses of VIS410 when administered in combination with oseltamivir in hospitalised subjects with influenza A infection. (3) The proportion of subjects with adverse events following administration of VIS410	NCT03040141
MEDI8852+ oseltamivir	IIa	Adults with uncomplicated influenza, age 18 to 65 years (*n* = 126)	Intravenous MEDI8852 750 mg or MEDI8852 3,000 mg or matching placebo on day 1 in combination with oseltamivir 75-mg BID from day 1 to 5	(1) Number of participants with influenza symptoms on day 1 to 10 and then day 10 to 13. (2) Number of participants with treatment-emergent adverse events, treatment-emergent serious adverse events or treatment emergent adverse events of special interest	NCT02603952
MHAA4549A + oseltamivir	II	Patients with severe influenza A infection, age 18 years or older (*n* = 168)	Intravenous MHAA4549A 3,600 mg or 8,400 mg or matching placebo in combination with oseltamivir 75 or 150-mg BID for 5 days	(1) Percentage of participants with adverse events. (2) Number of participants with anti-therapeutic antibodies to MHAA4549A during and following administration of MHAA4549A. (3) Time to normalisation of respiratory function	NCT02293863
Celecoxib	III	Severe influenza A, age 18 years or older (*n* = 107)	Celecoxib 200 mg daily, or a matching placebo, in combination with oseltamivir 75-mg BID for 5 days	Mortality rate at 28 days mortality from hospitalisation	NCT02108366
Diltiazem	II	Severe influenza A, age 18 years or older (*n* = 300)	Diltiazem 60 mg, 3 times a day, in combination with oseltamivir 150-mg BID, for 10 days. Or a placebo of diltiazem with oseltamivir, 150-mg BID, for 10 days	Percentage of alive patients without detection of influenza A virus by RT-PCR in nasopharyngeal swabs	NCT03212716
Nitazoxanide	III	Acute uncomplicated influenza, age 13 to 65 years (*n* = 1941)	Nitazoxanide 300 mg in combination with oseltamivir 75-mg BID for 5 days or either drug alone at the same dose administered with placebo	Time to resolution of all clinical symptoms of influenza as reported by the subjects	NCT01610245
Clarithromycin	II	Hospitalised pediatric influenza patients, 1 to 18 years (*n* = 54)	Clarithromycin (500 mg), naproxen (250 mg), oseltamivir (adjusted by weight 30 to 75 mg) combination therapy to that of oseltamivir treatment alone	Time to resolution of fever and decrease of the PRESS	NCT04315194

AUC, Area Under the Curve; BID, bis in die (twice a day); NAI, neuraminidase inhibitor; NEWS2, national early warning score 2; PRESS, Pediatric Respiratory Severity Score; PRO, patient-reported outcome; qRT-PCR, quantitative reverse transcription PCR; SOC, standard of care; TID: ter in die (1 times day).

### Favipiravir in vitro and preclinical studies

Synergistic interactions between favipiravir and the NAIs have been shown in vitro [63]. Smee and colleagues showed in vivo synergy in mice infected with A(H1N1) and A(H3N2) when treated 24 hours postinfection with favipiravir + oseltamivir showed synergy, but there was only a small improvement in survival with A(H5N1) challenge [64].

Delayed (96 hours postchallenge) treatment with favipiravir and peramivir twice daily for 5 days in mice infected with A/California/4/2009 (A(H1N1pdm09)) showed synergistic interactions with favipiravir at 20 mg/kg combined with 0.025, 0.05, or 0.1 mg/kg/day of peramivir [65]. The combination of peramivir + favipiravir was beneficial at suboptimal doses and led to greater improvement for survival and body weight, but not for lung viral titres relative to monotherapy with higher doses [65]. Favipiravir + peramivir treatment in mice infected with oseltamivir-resistant A(H1N1pdm09) virus showed monotherapy with either drug resulted in severe weight loss in all mice [66]. The combination was synergistic at higher doses of favipiravir (20 and 40 mg/kg) to reduce mortality. Favipiravir monotherapy (40 mg/kg) was as effective as favipiravir (20 mg/kg) + peramivir (50 or 100 mg/kg) in combination [66]. Delayed treatment of A(H5N1) infection with favipiravir (50 mg/kg/day) + oseltamivir (20 mg/kg/day) protected 100% of mice from mortality and reduced weight loss more effectively than monotherapy [67]. Several substitutions emerged following treatment in the PB1 gene, but these mutations did not change the in vitro drug susceptibility [67].

Treatment was commenced 48 hours postinfection with oseltamivir (20 mg/kg) and a low or high dose of favipiravir (20 or 40 mg/kg/day) twice daily for 5 or 10 days in mice immunosuppressed with cyclophosphamide treatment and infected with A(H1N1pdm09) [68]. A low dose of favipiravir + oseltamivir did not reduce mortality in mice compared to monotherapy; however, lung viral titres were lower on days 8 and 12 postinfection. A high dose of favipiravir (40 mg/kg/day) resulted in equivalent survival rates in all treatments (monotherapy and combination therapy) [68]. Oseltamivir + favipiravir reduced lung viral titres to a similar magnitude as favipiravir monotherapy, and both were more effective than oseltamivir alone [68].

### Favipiravir clinical studies

A Phase IIa, open-label trial tested pharmacokinetics of favipiravir and oseltamivir in combination in patients with severe influenza [39,69]. The dosing regimens were well tolerated; however, patients may require higher drug doses to achieve the desired threshold for favipiravir concentration in plasma [69].

A separate retrospective analysis from this study compared oseltamivir + favipiravir treatment (NCT03394209) to oseltamivir alone (40 and 128 patients, respectively (Table 2)) [69]. Both treatment groups had the same median time to clinical improvement; however, combination treatment reduced the number of severe outcomes compared to monotherapy [69]. Ten days posttreatment, the proportion of patients with no detectable viral RNA was 67.5% and 21.7% for combination and oseltamivir monotherapy, respectively [69].

In a case study of a severely immunocompromised child with influenza B virus infection, a combination of oseltamivir, zanamivir, and nitazoxanide failed to clear infection [70]. With an alternative treatment combination of favipiravir, oseltamivir and zanamivir, the patient tested negative for influenza for 2 months, after which influenza B was detected again [70]. The infection was cleared after further treatment with favipiravir + zanamivir for 2 weeks [70]. This combination failed to prevent the emergence of zanamivir resistance; the resistant virus represented a proportion of the virus selected following treatment [70].

### Pimodivir in vitro and preclinical studies

In vitro studies have shown synergy between pimodivir and oseltamivir, zanamivir, or favipiravir [71]. To our knowledge, in vivo studies have only assessed pimodivir monotherapy [71,72].

### Pimodivir clinical studies

A Phase Ia safety and pharmacokinetic study with pimodivir and oseltamivir shown no clinically relevant drug–drug interactions or safety concerns (Table 2) [73].

A Phase II double-blind trial in patients with uncomplicated influenza compared 3 treatment groups; pimodivir monotherapy at either 300 or 600 mg and the combination of pimodivir (600 mg) + oseltamivir (75 mg) (NCT02342249) (Table 2) [74]. The time to viral clearance relative to placebo based on qPCR was reduced by 31%, 13%, and 18% for oseltamivir + pimodivir, 300 mg pimodivir, and 600 mg pimodivir, respectively [74]. The primary endpoint of reduction of viral load was met, and the study was terminated early [74]. Viruses with PB2 substitutions such as S324K/N/R, F325L, or reduced susceptibility to pimodivir were detected in 6.9%, 10.5%, and 1.8% of 300 mg pimodivir, 600 mg pimodivir, and pimodivir +oseltamivir patients, respectively [56].

A Phase II clinical trial compared combination treatment with pimodivir + oseltamivir versus oseltamivir alone in hospitalised patients with influenza A(NCT02532283) (Table 2) [38]. Pharmacokinetics, the primary endpoint, in elderly and nonelderly patients was the time to patient-reported symptom resolution; for pimodivir + oseltamivir was 72.45 hours versus 94.15 hours for the oseltamivir group [38]. Viral clearance was faster in the pimodivir + oseltamivir group than the oseltamivir monotherapy group (72 and 96 hours, respectively) [38].

Two Phase III placebo-controlled trials were initiated for pimodivir, one was in hospitalised adolescents and adults (NCT03376321) and the second was in high-risk outpatients (NCT03381196) (Table 2). The study in hospitalised patients was terminated early based on interim analysis, but the results have not been published yet. Summary results for both trials are available on the ClinicalTrials.gov website. The primary outcome was clinical status based on a hospital recovery scale; there was no difference between pimodivir and SOC treatment (based on the local practice; either antivirals or supportive care) for avoiding hospitalisation (48.03% and 47.59%, respectively) or for other measures on the recovery scale. The time to hospital discharge in the pimodivir group was 113 hours and 108 hours for SOC. The viral load over time, measured by qPCR, showed no significant differences between the treatment groups. The viruses from study participants were also analysed for “mutations of interest” in the NA and PB2 genes that are known to confer drug resistance. Although the specific amino acid changes are not listed, participants treated with pimodivir + SOC had no mutations of interest in the NA gene, and 1.3% (2/159) participants had mutations of interest in the PB2 gene. In patients treated with SOC, 1.9% (3/159) had mutations of interest in the NA gene and none in the PB2.

The second Phase III study was conducted in adolescent, adult, and elderly patients at high risk of developing complications (NCT03381196) where the primary endpoint was the resolution of influenza-related symptoms. The median time was 92.62 and 105.13 hours in the pimodivir and SOC treatment groups, respectively. Mutations of interest (not disclosed) in the pimodivir + SOC treatment group occurred in 5.6% (4/71) of viruses isolated from participants while none occurred (0/108) in the group treated with SOC.

### Concluding remarks on polymerase inhibitors

Preclinical studies in mice show that the combination of baloxavir and favipiravir or oseltamivir does not have an added benefit over monotherapy for reduction of lung titres and weight loss, but does for mortality. However, the combination of an NAI with baloxavir may reduce the selection of resistant viruses particularly the frequency of PA/I38X substitutions. Preclinical studies suggest that favipiravir can be synergistic with NAIs to reduce mortality and weight loss, but the reduction in lung titres is variable.

In immunocompromised patients, the combination of baloxavir and NAIs provided no clinical benefit over monotherapy, but did reduce the duration of viral shedding. Pimodivir showed early promise as an influenza antiviral treatment, with less safety concerns than favipiravir and encouraging results in Phase II trials. The termination of the pimodivir Phase III trials due to lack of efficacy based on primary endpoints is a disappointing outcome, but it will be interesting to see if pimodivir continues to be investigated in the future in different patient cohorts or in other antiviral combinations. Interestingly, favipiravir clinical trials had similar results to baloxavir, where combination treatment with oseltamivir did not improve the time to alleviation of symptoms but reduced the duration for which virus could be detected.

The combination of baloxavir and NAIs, including those hospitalised with severe influenza, warrants further investigation. However, the emergence of dual resistant viruses must be monitored especially in those that are severely immunocompromised. For pimodivir, the incidence of mutations of interest was low in both treatment groups; however, further information on the amino acid changes will be useful.

### Monoclonal antibodies (mAbs)

There are several mAbs and broadly neutralising antibodies (bnAbs) targeting conserved regions on the influenza hemagglutinin (HA) or NA in clinical development. These antibodies may be useful in combination with direct-acting antiviral drugs to treat drug-resistant viruses or in severe influenza.

### VIS410

VIS410 is an HA stem-targeted mAb (IgG1) that has been assessed as monotherapy in a Phase I (NCT02045472) and 2 Phase IIa clinical trials (NCT02989194 and NCT02468115) (Table 1). An early preclinical study of VIS410 by Tharakaraman and colleagues showed that a low dose of VIS410 (5 mg/kg) + oseltamivir (10 mg/kg daily) resulted in significantly less weight loss than either monotherapy and a high-dose combination (VIS410 20 mg/kg + oseltamivir 60 mg/kg) resulted in faster weight loss recovery than monotherapy. Infection of mice with A/Shanghai/2/2013 (A(H7N9) and VIS410 + oseltamivir alone or in combination showed that VIS410 and combination treatment provided similar protection against weight loss. A Phase II clinical trial (NCT03040141) is listed on the ClinicalTrials.gov website, to evaluate the combination of oseltamivir + VIS410 for hospitalised adult patients that require oxygen. The primary clinical outcome was based on a 7-level ordinal recovery scale (Tables 1 and 2). Although the study is complete, results are not yet available.

### MHAA4549A

MHAA4549A is a mAb that targets a highly conserved HA stalk epitope on the HA stalk of influenza A viruses and was synergistic in combination with oseltamivir in vitro [75]. There are 3 Phase II clinical trials listed on ClinicalTrials.gov website involving MHAA4549A, 2 are monotherapy studies (NCT02623322 and NCT01980966), and 1 is a combination study with oseltamivir in severely ill patients (NCT02293863) (Table 1).

The trials for uncomplicated influenza and hospitalised patients with severe influenza both showed that MHAA4549A was present in the serum of patients, and there were no negative pharmacokinetic interactions when MHAA4549A was administered in combination with oseltamivir [76,77]. The combination study in hospitalised patients had a primary endpoint of time to cessation of supplemental oxygen support, for which there was a nonsignificant decrease compared to placebo (4 days) in the 3,600 mg (2.8 days) and 8,400 mg (2.7 days) groups [78]. Other endpoints such as the time to ICU discharge, 30 day all-cause mortality and duration of viral shedding showed nonsignificant differences between the treatment groups [78].

### CF-404

Vigil and colleagues investigated CF-404, a 3 mg/kg triple combination that included 3 anti-HA stalk bNAbs that target influenza A group 1 (TRL053/CF-401), group 2 (TRL579/CF-402), and influenza B (TRL849/CF-403) viruses [79]. CF-404 protected mice infected with A(H1N1), A(H3N2), or influenza B (B/Victoria and B/Yamagata lineages) viruses from death and weight loss. The effectiveness of monotherapy or dual combinations with the antibodies that constitute the triple combination formula was not shown, likely because the aim of the paper was to achieve broad-spectrum activity rather than to demonstrate synergy. Nonetheless, a triple bNAb combination that is effective against influenza A and B viruses is likely to be of clinical value.

### Favipiravir + CR9114 + F3A19

Immunocompromised (nude) mice were infected with A/California/04/2009 (A(H1N1pdm09)) and treated with a triple combination of 2 mAbs targeting the receptor binding site (RBS) of HA (F3A19, 1 mg/kg every 3 days for 14 days), HA stem (CR9114, 5 mg/kg every 3 days for 14 days), and favipiravir (100 mg/kg daily for 28 days) [80]. All mice treated with a triple combination had greater survival than monotherapy. Virus titres were lower in favipiravir combinations, compared to monotherapies. Although in an immunocompromised model, viruses with reduced susceptibility to favipiravir were not identified posttreatment, and some HA mutations were found but not deemed to be antibody escape mutants based on their location on the HA protein [80].

### 1D2 + 1F2 (influenza B anti-HA and NA)

Influenza B/Brisbane/60/2008 was used to study infection of immunosuppressed mice and subsequent treatment with anti-influenza B mAbs [81]. Combination treatment with 1D2 (anti-HA, 10 mg/kg) or 1F2 (anti-NA, 1 mg/kg) was compared to monotherapy at 24, 48, and 72 hours postinfection [81]. Combination treatment administered at 48 hours postinfection was more effective than either monotherapy to prevent death and extended mean survival time, but was equal to monotherapy for both parameters when administered at 24 and 72 hours postinfection [81]. There was no difference in virus lung titre of mice following monotherapy or combination treatment, but less immunopathology and lung damage from viral infection occurred in the combination treatment group [81]. No viral escape mutants were identified from HA and NA gene sequencing [81].

### CT-P27

CT149 (influenza bNab; HA fusion domain binding) has previously been shown to neutralise most group 2 and some group 1 influenza viruses and showed encouraging efficacy in vitro and in vivo [82]. The authors further developed another antibody to be used in combination with CT149 (CT120, influenza bNab; to broaden the spectrum of viruses neutralised [83]. Mice treated with the resultant bNAb, CT-P27, had 90% survival when infected with A/California/04/2009 (A(H1N1pdm09)) or A/Philippines/2/1982 (A(H3N2)) at the 2 higher doses tested (15 or 30 mg/kg) [83]. Prophylaxis with CT-P27 at 30 mg/kg; administered 14 days prior to infection had 100% and 90% survival with A/California/04/2009 and A/Philippines/2/1982, respectively [83]. CT-P27 (1.875 or 3.75 mg/kg) in combination with oseltamivir (20 mg/kg) had improved compared to monotherapy at all doses, with a 100% survival at the higher dose [83]. There are 2 clinical trials with CT-P27 monotherapy listed on the ClinicalTrials.gov website (NCT02071914 and NCT03511066), but results are not yet available.

### MEDI8852

MEDI8852 is an HA stalk mAb that is active against all 18 influenza A subtypes. For H7N9 and H5N1 infection in ferrets, antiviral treatment was commenced 8 or 24 hours following infection with A/Anhui/01/2013 (H7N9) with MEDI8852 (25 mg/kg, single dose), oseltamivir (12.5 mg/kg BID 5 days), or oseltamivir + MEDI9952 in combination [84]. Treatment 24 hours postinfection showed combination therapy was more effective than either monotherapy for reduction in body weight loss [84]. In a dose-ranging experiment with H5N1 virus challenge in ferrets, the combination of MEDI8852 with oseltamivir improved survival rates and protection from weight loss more than either monotherapy alone [84]. A Phase IIa trial for treatment of uncomplicated influenza enrolled 128 participants to receive a low (750 mg) or high (3,000 mg) dose of MEDI8852 with oseltamivir (75 mg) or oseltamivir monotherapy or high-dose MEDI8852 alone (Table 2) [85]. The time to resolution of symptoms was similar in each group: 106.75, 128, 138.10, and 106.75 hours, respectively, and there was little difference in viral shedding measured by qRT-PCR [85]. Sequencing of the NA gene showed that none of the samples have known resistance mutations to the NAIs [85]. Six HA mutations were identified in patient samples, but none of them corresponded to MEDI8852 binding sites or have altered susceptibility to MEDI8852.

### Concluding remarks on mAbs

A diverse range of mAbs have been investigated in preclinical trials, most showing encouraging results and low selection of escape mutants. mAbs treatment for severe influenza is of interest; however, at the present time, few have progressed to clinical trials. Given the mechanism of action, mAbs have the potential to provide synergistic benefits for the treatment of influenza with antiviral drugs that directly target the replication cycle of the virus.

### Host-targeted therapies, anti-inflammatory drugs, and immunomodulators

#### Nitazoxanide

Nitazoxanide is a repurposed, host-targeted antiparasitic drug that has also been shown to have broad-spectrum antiviral activity against influenza viruses (Table 1). In vitro drug synergy with nitazoxanide and either oseltamivir or zanamivir has been identified against A(H1N1) and A(H5N9) viruses [86,87]. In ferrets, the combination of oseltamivir + nitazoxanide administered as prophylaxis 2 hours prior to infection had significantly lower virus shedding and no virus in the lower respiratory tract, compared to either monotherapy [87]. However, the effectiveness was lower when antiviral treatment was commenced 24 hours postinfection [87]. A further study comparing oseltamivir + nitazoxanide combination therapy in ferrets infected with a mixture of 1% oseltamivir resistant (NA/H275Y) and 99% wild-type drug-sensitive virus (A/Perth/261/2009 and A/Perth/265/2009) showed no difference in the selection of oseltamivir resistant NA/H275Y virus and viral titres in nasal washes compared to monotherapy [88]. A Phase III clinical trial (NCT01610245) with oseltamivir + nitazoxanide combination treatment has been completed, but results are not yet available (Tables 1 and 2).

#### Diltiazem

Diltiazem, a calcium channel blocker, was predicted by in vitro transcriptional profiling to have an antiviral effect against influenza [89]. A retrospective study was completed by Wang and colleagues to analyse patients in a de-identified medical database. Data for adult patients (*n* = 302) treated with oseltamivir were extracted; of these, the author identified 36 patients who had also received diltiazem during their hospital admission. This analysis showed that there was a decrease of in-hospital mortality in those treated with oseltamivir and diltiazem. A Phase II clinical trial (NCT03212716) evaluating oseltamivir + diltiazem versus placebo was initiated (Table 2). The primary outcome is mortality in patients with severe influenza; results of the Phase II clinical trial will be of interest when they become available [90].

#### Celecoxib

Celecoxib is a nonsteroidal anti-inflammatory drug that inhibits COX-II and is of interest to prevent excessive inflammation that can result in severe lung disease. The combination of celecoxib with antisense RNA oligonucleotides targeting the PB2 gene significantly reduced the viral load (based on qPCR) and inflammatory markers in mice [91]. Survival rate and inflammatory markers were improved in H5N1 virus infected mice treated at 48 hours postinfection with a combination of zanamivir (3-mg BID), celecoxib (2 mg once a day), gemfibrozil (lipid regulating drug; 1 mg once a day), and mesalazine (aminosalicylate used for inflammation; 1 mg once a day) [92]. The triple drug combination improved lung viral titres, which was with combination treatment and zanamivir monotherapy [92]. Based on these results, a Phase III clinical trial was initiated for severe influenza A infection (NCT02108366) (Table 2), a significant reduction in 28-day mortality (primary endpoint) in the oseltamivir + celecoxib treatment group compared to oseltamivir alone was shown [93].

### High mobility group box-1 (HMGB1)

HMGB1 is a nuclear protein that regulates gene transcription when released from immune cells or from necrotic cells. This protein is being investigated as a therapeutic target to reduce elevated inflammatory cytokine and chemokine levels during infection [94]. Treatment with anti-HMGB1 was studied for severe influenza in combination with NAIs for A/Puerto Rico/8/34 infection in mice [94]. Delayed treatment improved survival in mice and there was less histopathology and neutrophil and macrophage aggregation compared to monotherapy, but anti-HMGB1 alone did not inhibit viral replication [94].

### Anti-inflammatory and immunomodulators concluding remarks

Since the resolution of symptoms is a key endpoint in clinical trials for the treatment of influenza, the addition of an anti-inflammatory drug may provide faster recovery of symptoms. For direct-acting influenza drugs such as baloxavir and pimodivir, the combination with an anti-inflammatory drug may be an interesting area of research. In addition, a host-targeted drug in combination with influenza antiviral drugs is of interest particularly if the selection of drug-resistant viruses is reduced.

### Antiviral and antibiotic combinations

#### Azithromycin

Azithromycin (broad-spectrum macrolide antibiotic; 100 mg/kg single dose) + oseltamivir (10 mg/kg once daily) treatment in mice at 48 or 72 hours postinfection in mice infected with A/California/07/2009 (A(H1N1pdm09)) showed no improvement in lung viral titres, inflammatory cytokine levels, or mouse survival rates compared to monotherapy [95]. A retrospective study (Table 2) showed patients infected with influenza A or B and treated with oseltamivir + azithromycin (102 patients) required less oxygen support and had shorter hospitalisation time and compared to oseltamivir alone (227 patients) [96]. An open-label, randomised, multicentre trial (107 patients) showed the combination of oseltamivir and azithromycin in patients had no difference, relative to monotherapy, for inflammatory markers or the resolution of influenza related symptoms [97].

#### Clarithromycin

Clarithromycin is also a broad-spectrum macrolide antibiotic. Mice were treated with a triple combination of flufenamic acid (nonsteroidal anti-inflammatory drug, 50 mg/kg, for 3 days) + clarithromycin (50 mg/kg BID for 3 days) + zanamivir (100 mg/kg BID for 4 days) following infection with A/415742Md/Hong Kong/2009 (A(H1N1)) [98]. The triple drug combination was the most effective treatment to prevent lethality and body weight loss [98]. A prospective, single-blind study in children showed resolution of fever was halved with clarithromycin-naproxen-oseltamivir treatment compared to oseltamivir monotherapy and had transiently greater reduction in viral titre. However, the length of hospitalisation was similar in the 2 treatment groups [99]. A Phase IIb/III, open label study in hospitalised patients, evaluated clarithromycin + naproxen + oseltamivir and the 30 day mortality (primary endpoint) was reduced compared to oseltamivir alone, as was the length of hospital stay and viral titres (Tables 1 and 2) [100].

#### Concluding remarks on antiviral and antibiotic combinations

Secondary bacterial infections following influenza infection have the potential to lead to more severe outcomes, especially in high-risk patients. The combination of an antiviral drug with an antibiotic is hypothesised to prevent the progression of influenza illness to secondary bacterial infection that may require further hospitalisation or lead to more severe outcomes. However, the proposed benefit of the use of antibiotics should be balanced against the risks of the selection of antibiotic resistance.

## Conclusions

The licensure of antiviral drugs like baloxavir marboxil with novel mechanisms of action will expand the potential for combinations of drugs for the treatment of influenza. Clinical trials with combination therapy must include diverse patient cohorts, especially patients at high risk of complications who may accrue the greatest benefit from such therapy. However, diversity of patient cohorts adds complexity to our understanding of the effectiveness of combination therapies. Host-targeted or adjunctive therapies combined with direct-acting antiviral drugs are of great interest, but more studies in the best use of such treatments is required. Finally, antiviral resistance is a major concern for direct-acting antiviral drugs, and there is some evidence to suggest that combination treatment may reduce the likelihood of selecting resistant virus.
